# Key Considerations for Successful Risk Communication and Community Engagement (RCCE) Programmes During COVID-19 Pandemic and Other Public Health Emergencies

**DOI:** 10.5334/aogh.3119

**Published:** 2020-11-17

**Authors:** Laston Gonah

**Affiliations:** 1 Community Medicine Department, Faculty of Medicine, Midlands State University, Gweru, ZW

## Abstract

Risk communication and community engagement (RCCE) is a key pillar in public health emergency response that ensures accurate health information sharing, adoption of protective behaviours by the affected people, and collaborative participation by all stakeholders, including of the local community structures. The success of RCCE programmes rely on strong partnerships and engagement among affiliated groups; clear programme plans and guidelines; establishment of well-laid down coordination structures; and clear measures for reporting and documentation of programme activities. RCCE activities during public health emergencies must put more emphasis on strengthening local structures and communities to ensure active participation of communities in interrupting disease transmission.

## Background

The World Health Organization identified Risk Communication and Community Engagement (RCCE) as one of the eight key pillars in responding to the COVID-19 Public Health Emergency of International Concern [1]. Risk communication helps to ensure exchange of accurate information among healthcare workers and authorities, and between healthcare workers and the population at risk, with the aim of achieving improvement in the knowledge and understanding of the disease (modes of spread, signs and symptoms, preventive and treatment measures) and adoption of appropriate protective or treatment behaviours [1,2]. Community engagement brings together all the groups of people that are affiliated by geographic proximity, special interest and similar risk situations or circumstances, working collaboratively in addressing issues affecting their health and well-being [1].

RCCE is often considered the least important of the public health emergency response pillars, yet it is actually key in breaking the chain of infection during a disease outbreak. RCCE has become increasingly important during COVID-19 response in promoting non-pharmaceutical interventions (NPIs) to interrupt disease transmission, in the face of delayed vaccine development process. Existing literature has pointed to the importance of RCCE in public health emergency preparedness and response, both in general and with particular focus on specified disease outbreaks [2,3,4,5,6]. A quick, simplified guide to key considerations for ensuring successful RCCE programmes is worthwhile, to guide present and future RCCE efforts in public health emergency response efforts. Responding to public health emergencies, like the current COVID-19 pandemic, requires a quick but well-structured approach to contain or mitigate the disease outbreak. This paper details key considerations that are important for successful RCCE programmes during a public health emergency.

## Components of successful risk communication and community engagement programmes

**Figure 1:**
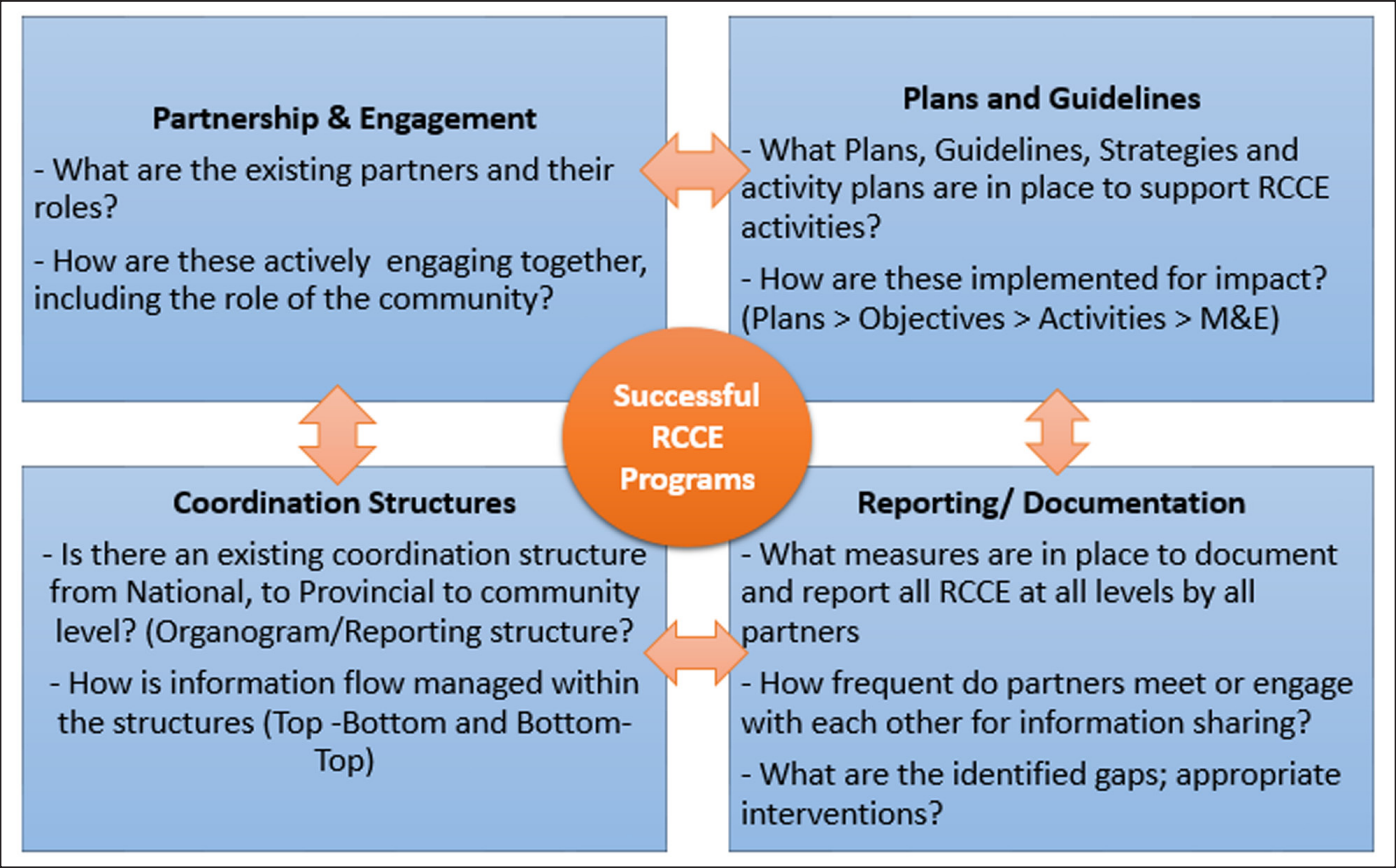
Key considerations for successful RCCE programmes.

The success of RCCE programmes relies on strong partnerships and engagement; clear programme plans and guidelines; establishment of well-laid down coordination structures; and clear measures for reporting and documenting programme activities, and these factors are inextricably linked in successful RCCE programmes during public health emergencies (Figure 1).

### 1. Partnership and engagement

A public health emergency often brings together various sectors responding to the emergency under several response pillars, including RCCE [1,4]. In as much as partnership is important in responding to public health emergencies, it is how the work by various partners is coordinated that matters the most. Mapping of all the partners involved in RCCE in a particular country or province or district, or area should be emphasised as a key priority in any response plan [1]. All the partners must be identified, their roles clearly defined and consistently updated from time to time, to identify areas for improvement or support. In RCCE, various activities are involved, including provision of technical and implementation support; funding and resource mobilization; advocacy; production and supply of materials and equipment, among others. In most emergencies, parallel competing programmes are common if there is no coordination of partner activities and roles. For RCCE, the main challenge is that, parallel and uncoordinated programmes by different partners has the potential to duplicate interventions unnecessarily resulting in inefficient use of resources; or implementation of conflicting interventions.

The government body overseeing the response should ensure that all partners are identified, and a plan is put in place where they come together to collaborate through such activities as update meetings, for sharing of information, or that their various activities are documented and consolidated into a single report. It must always be remembered that the primary goal of intervening in public health emergency is to contribute in the interruption of transmission or the ending of the public health emergency, and this happens especially when there is proper coordination of partner activities within the affected area. While RCCE plays an important role in public health emergency response, poorly defined stakeholder roles can prove to be counterproductive on the achievement of the primary goal.

### 2. Plans and guidelines

To most countries, the COVID-19 came as a surprise, when the health systems were not prepared to deal with the outbreak, in terms of financial, human, and material resources. RCCE plays a key role, from the moment an outbreak is anticipated, to the time the outbreak starts and peaks, and up to the moment the outbreak recedes and in post-outbreak era, in strengthening community participation and action in outbreak control and in addressing various information needs of the affected communities, for adoption of recommended behaviours.

Since RCCE involves many areas and partners, as mentioned above, it is very easy and common to get things wrong in the response if activities are not planned and guided. Planning forms key component of RCCE programme implementation since it details the contextual problem(s) or gaps at hand, the set objectives, the planned activities and assigned roles and timelines for implementation of the activities, and well as the monitoring and evaluation parameters to assess progress over time. Strategic plans, activity plans and related guidelines, must be informed by the prevailing situation and resource capacities at hand, for meaningful response to the public health emergency, and must be continuously updated from time to time as the situation unfolds. The input or contribution of technical and implementation partners in developing or updating plans and guidelines is key in ensuring timely and successful implementation of the planned interventions or activities, as it creates the spirit of programme ownership and responsibility among partners.

### 3. Coordination structures

Coordination is another key component required for successful RCCE programmes, some reasons which have been addressed under partnership and engagement above [1,2]. The most important aspect of outbreak response in an emergency is information flow from the top to the lowest level, and vice versa, within the affected communities. Therefore, the establishment of a coordination structure for RCCE from the highest level to the lowest level ensures that the much needed information and feedback is exchanged as fast and as efficiently as possible. RCCE activities, and any other programme activities, where proper coordination structures are not set and defined at all levels, results in disjointed structures and lack of accountability, and therefore lead to a poor response to the public health emergency. For instance, countries must ensure that structures (with well-defined roles) for RCCE are established from Country level to Provincial, to district, to sub-district, to ward, and to community level, or their equivalences. When this is done, community concerns or feedback is efficiently transferred to the top for immediate appropriate attention, and relevant support will timeously reach to the community as required.

### 4. Reporting or documentation

As the old adage goes, “what is not documented is not done”. Unfortunately, most activities and best practices in an emergency go unnoticed simply because they are not documented and reported. Lack of reporting emanates from any or all of poorly defined or disjointed coordination structures; poor activity planning; and poorly defined partner roles, among others. In the COVID-19 response, a lot of good work has been done by various entities, especially on RCCE, but due to the reasons mentioned above, most of the activities and best practices have not been documented and reported, presenting a picture of little to no activity done or implemented. RCCE principles need to set standards for documenting and reporting activities and experiences, with clearly defined monitoring and evaluation indicators, to substantiate that RCCE plays a key role in outbreak response, just like any other key pillars of emergence response.

## Conclusions

RCCE is an essential component of public health emergency or disease outbreak response that needs to be redefined to ensure that mapping of partners; proper coordination structures; activity planning; and documentation and reporting of activities and experiences are prioritised for significant contribution to the overall public health emergency response.

## Figures and Tables

**Table:** 

IEC:	Information, Educational, Communication
COVID-19:	Corona Virus Disease 2019
NPIs:	Non-pharmaceutical Interventions (NPIs)
RCCE:	Risk Communication and Community Engagement
WHO:	World Health Organization
